# Decision Support System for Liver Lesion Segmentation Based on Advanced Convolutional Neural Network Architectures

**DOI:** 10.3390/bioengineering9090467

**Published:** 2022-09-13

**Authors:** Dan Popescu, Andrei Stanciulescu, Mihai Dan Pomohaci, Loretta Ichim

**Affiliations:** Faculty of Automatic Control and Computers, University POLITEHNICA of Bucharest, 060042 Bucharest, Romania

**Keywords:** decision support system, liver lesions, computed tomography, neural networks, semantic segmentation, decision fusion

## Abstract

Given its essential role in body functions, liver cancer is the third most common cause of death from cancer, despite being the sixth most common type of cancer worldwide. Following advancements in medicine and image processing, medical image segmentation methods are receiving a great deal of attention. As a novelty, the paper proposes an intelligent decision system for segmenting liver and hepatic tumors by integrating four efficient neural networks (ResNet152, ResNeXt101, DenseNet201, and InceptionV3). Images from computed tomography for training, validation, and testing were taken from the public LiTS17 database and preprocessed to better highlight liver tissue and tumors. Global segmentation is done by separately training individual classifiers and the global system of merging individual decisions. For the aforementioned application, classification neural networks have been modified for semantic segmentation. After segmentation based on the neural network system, the images were postprocessed to eliminate artifacts. The segmentation results obtained by the system were better, from the point of view of the Dice coefficient, than those obtained by the individual networks, and comparable with those reported in recent works.

## 1. Introduction

The liver is the largest organ in the human body and is responsible for multiple essential functions. It accounts for around 2% of an adult’s body weight and is a unique organ due to its dual blood supply: the portal vein and the hepatic artery. Bile production plays a vital role in the excretion of substances that cannot be processed by the kidneys, while also aiding in the absorption and digestion of lipids. The liver’s metabolic functions consist of fat-soluble vitamin storage and the decomposition of xenobiotics, such as various toxins and carcinogens. Lastly, the liver is also responsible for the processing of bilirubin, which is one of the products of hemoglobin breakdown [1]. Given its essential role in body functions, liver cancer is the third most common cause of death from cancer, despite being the sixth most common type of cancer worldwide [2]. Following advancements in medicine and image processing, medical image segmentation methods are receiving a great deal of attention.

In practice, liver lesions are observed and diagnosed by comparing patterns that appear when contrast imaging techniques are used. One of the most widely used methods for liver disease diagnosis is computed tomography (CT). The liver has soft tissue density, similar to the tumors that affect it, so plain CT images lack the contrast necessary to distinguish between areas of interest. To combat this setback, an intravenous contrast substance is administered to the patient. The contrast reaches the tumor in a different pattern compared to the liver parenchyma. The diagnosis is made by comparing the contrast enhancement pattern of the lesions with the hepatic structure in the different acquisition phases post-contrast administration (arterial, portal-venous, and interstitial phases) [3].

Researchers have proposed several computer-based systems to aid radiologists in interpreting CT images. Computer-aided detection (CAD) systems are used to analyze and report areas in images that may be of interest. On the other hand, CAD systems not only detect the regions of interest in CT images but also present the likelihood that the area is a specific type of lesion, for example, malignant or benign. The main objective of both types of systems is to reduce the number of false negatives caused by human error. CAD systems have been developed to search for and mark similar features in digital images that radiologists would look for when performing an investigation [4]. The development of these systems requires the integration of multiple aspects of computer vision, such as image processing, pattern matching, and artificial intelligence. The workflow necessary for hepatic CAD consists of the following stages: pre-processing, segmentation, feature extraction, and selection and classification. Depending on the method used for the feature extraction step, these computer-aided diagnosis tools can be classified as conventional- or deep-learning-based [5]. 

In the case of liver diseases, automated diagnostic techniques have seen a great deal of interest, which is reflected by the steady increase in research papers on this subject. The implementation of these methods will reduce the necessity of manual segmentation, which is prone to human error and inter-reader variability. A correct segmentation and volumetric evaluation of the hepatic structure helps in deciding on future management, including the evaluation of oncological disease burden, liver remnant volumes in hepatic resection and living donor transplantation, and others. For example, in the case of hepatectomy for liver lesions, it is important to correctly assess the functional liver remnant (FLR) after resection to ensure that enough hepatic parenchyma is left to maintain liver function. At least 20% of the functional liver volume must remain in the absence of liver disease, while at least 30–40% must be left in the case of liver disease according to severity [6]. These measurements are usually done manually by trained radiologists and require precise delineation of the liver structure, hepatic segments, and lesions that need to be resected, which is time-consuming. Moreover, the ability to segment liver lesions is essential for the future development of classification task models by classical machine learning radiomics or by using deep learning techniques and will provide an important assisting tool for hospital multidisciplinary teams. 

An important aspect that drives this initiative is the presence of public datasets, such as LiTS17 [7], which contain anonymous CT examinations along with reference segmentation masks provided by certified radiologists. Other datasets available online are the 3DIRCAD [8] with CT imaging, the CHAOS [9] with both CT and MRI imaging, and the MSD dataset with CT imaging [10]. The existence of these online databases allows independent researchers who do not have any affiliation with medical institutes to perform research and advance technology in this field. 

In this context, this paper proposes a system based on new trends in the use of neural networks for the segmentation of liver and liver lesions. The objectives (O) and novelties (N) of the paper are listed below.

▪ O1: Provide an overview of the existing systems used in biomedical imaging, specifically liver and tumor segmentation methods, which achieved high scores in competitions and provide a starting point for developing a new system.
▪ O2: Provide detailed descriptions of the methods used to develop a segmentation model, starting with efficient convolutional neural networks.
▪ O3: Implementation of a decision fusion system with higher performance for liver and tumor segmentation. This is the main objective and has the following novelties:
  − N1: The method of establishing weights for individual neural networks.
  − N2: The method of combining individual decisions to obtain global decisions regarding segmentation (moving from individual intelligence to collective intelligence).
  − N3: The transition from classification to semantic segmentation.
  − N4: The method of learning the system in two stages (individual networks and global systems), each in two successive phases (for the segmentation of the liver and for the segmentation of the lesions).
▪ O4: Comparative analysis of the proposed system results and other relevant implementations presented in the literature.

All the codes and implementations used for this paper will be made publicly available under an open-source license so that others can verify, reproduce, and extend this work.

## 2. Related Works

The goal of this section is to provide an overview of technologies based on artificial intelligence and, especially, neural networks used in the last period in medical image segmentation and the diagnosis of liver and tumors into the liver. 

One method often used in medical imaging to detect tumors has been to break them down into patches and analyze them. Such a study explored tumor identification using patch analysis [11]. Before feeding the images to a convolutional neural network, a Gaussian filter was applied to remove noise. To classify each pixel, a patch of 17 × 17 pixels around it was passed through a CNN composed of five convolutional layers, two fully connected layers, which resulted in a classification output of ‘tumor’ and ‘non-tumor’. The method achieved a Dice Similarity Coefficient (DSC) of 80.06 ± 1.63%, which placed it above traditional machine learning techniques, namely AdaBoost, Random Forests, and Support Vector Machine (SVM). However, the method presented encountered difficulties when segmenting tumors of heterogeneous intensity or that had fuzzy borders. Patch analysis methods work in some cases, but they have drawbacks. For example, using a patch size that is too large requires additional max-pooling layers, which in turn causes the localization accuracy to decrease. Reducing patch size increases localization accuracy but, in turn, decreases contextual information.

An improvement to CNNs used in medical image analysis was brought about by the development of the U-Net [12]. Frequently, in medical applications, it is not enough to classify an image; instead, pixel-wise classification is necessary. U-Net works as an encoder–decoder sequence. To demonstrate the capabilities of this network, it was trained and tested for three different segmentation tasks: segmentation of neural structures from electron microscopy images, and two different cell tracking problems with images acquired by light microscopy. Because a small set of training data was used, data augmentation techniques, such as shifting, rotation, and elastic deformation, were employed to prevent overfitting and to teach the network the desired invariance needed for medical image segmentation. Across all three tests, the U-Net achieved better segmentation metrics than the other networks commonly used in image segmentation. An improved U-Net architecture proposed in [13] addresses the issue of low contrast between the liver and surrounding tissue, image noise, and differences in organ shape. The modification brought to the neural network consists of the method of transferring feature maps from the encoder to the decoder. This method is based on using the improved U-Net for initial liver segmentation and then employs a graph-cut algorithm to refine the segmentation from the probability maps. The experiments evaluating this network were performed on LiTS17, a liver segmentation competition dataset containing 131 sequences. 

The authors in [14] proposed a three-dimensional dual-path multi-scale convolutional neural network (TDP-CNN), followed by conditional random fields (CRF), which refine the segmentation result. This method takes advantage of the fact that CT images are usually taken as a series of slices through the body, thus providing 3D contextual information that is not used by 2D CNNs. As in other methods, the data were preprocessed by applying a Gaussian filter, normalization, and subsampling. To overcome the computational difficulties introduced by the use of 3D convolution and 3D feature maps, the initial volume is broken down into small segments, and only a few segments are input into the CNN at a time. Segments of different sizes but centered on the same voxel are used; hence, the term “multiscale”. The network is structured into two different paths. Segments with smaller image sizes but higher resolutions are processed by the local path and trained to capture features such as texture and contour. The bigger segments go through the global path, providing contextual and background information. The feature maps resulting from these paths are combined and used together in the final classification layers. The network classifies image regions into three labels: liver, liver tumor, and background. To correct mis-segmentation points, this implementation used a fully connected conditional random field. It can accurately segment liver and liver tumors from 3D abdominal CT images.

Another variation of the traditional U-Net architecture employs attention awareness and residual structures [15]. The standard blocks that perform encoding and decoding are wrapped in residual feed-forward structures, which address the problem of vanishing gradients in network training. Squeeze and Excitation blocks are introduced before the max pooling layers. These blocks suppress irrelevant areas of the image while highlighting significant ones. SE blocks consist of a global pooling layer, followed by an FC layer, a ReLU activation function, another FC layer, and then a sigmoid activation function. The output from the block has the same dimensions as the input, so a pooling layer is still necessary. Another difference from the original U-Net is how the transition from the encoder to the decoder is handled. Instead of using a standard convolution layer, the solution employs Atrous Spatial Pyramid Pooling (ASPP). Atrous convolution is a process that dilates the convolution kernel such that the elements from the original image are no longer neighboring pixels. By using multiple dilation factors for the atrous convolution, the context of the image can be captured at different scales. This enables the resulting feature map to contain richer semantic information. In this implementation, passing through the ASPP block doubles the number of features. The proposed system was evaluated using the LiTS17 training dataset. Several preprocessing steps were applied to the CT images. In this dataset, the network outperforms Attention U-Net, U-Net, and FCN. 

Combining residual attention-aware U-Nets with 3D convolutional neural networks, the authors in [16] proposed a novel architecture called RA-Unet, which can segment the liver and tumors from 3D volumetric images. Their implementation obtained competitive results when tested on the MICCAI 2017 LiTS17 dataset. Overall, the architecture consists of three individual stages. First, using a 2D residual attention-aware U-Net, a bounding box is placed around the liver in each slice of the volume to reduce redundant information for the next step. Next, the volume and the coarse bounding box are sent to a 3D RA-Unet, which defines a more precise volume of interest that contains only the liver. The volume is then sent to a second 3D RA-Unet, which extracts the tumor regions. Both the 2D and 3D versions of the RA-Unet share the same architecture, the only difference being the shape of the feature maps. Starting from the traditional U-Net structure, each downsampling and upsampling convolutional block is wrapped into a residual structure to combat the problem of vanishing gradients. The feature maps are passed through a residual attention-aware structure before being transferred to the decoder. This module is divided into two branches: the trunk branch processes the original features through a series of convolutional layers, while the soft mask branch selects features that are identical between feature maps and suppresses irrelevant noise. The soft mask branch consists of two encoder–decoder blocks, followed by two convolutional layers and a Sigmoid normalization layer. 

While previously presented methods rely on semantic segmentation of only one set of images with the purpose of highlighting the liver area and potential lesion areas, the authors in [17] proposed a system for tumor diagnosis that relies on the differentiation of liver masses in dynamic contrast agent-enhanced CT. This study used liver images acquired over three phases—noncontrast-agent enhanced, arterial, and delayed—to classify liver masses into five categories: classic hepatocellular carcinomas, malignant tumors other than HCCs, indeterminate lesions or mass-like lesions, hemangiomas, and cysts. This study focused on the classification of images of liver masses rather than determining the exact location of a lesion. The proposed network consists of a structure of two convolutional layers and one max-pool layer, a structure that is repeated three times. This is followed by three fully connected layers, which in the end output the probability for each type of liver mass. The patient images were captured in DICOM format, and then only the relevant slices were selected from each patient. To remove redundant information, a windowing function was applied to remove HU values outside the liver tissue range. The window ranges were adjusted to consider the differences between standard images and contrast-enhanced images. After the initial image preprocessing, data augmentation methods were used to make the system robust to disturbances, such as enlarging, rotation, parallel shift, and different levels of noise. Using these variations in the original images, 52 different datasets were obtained from the original data. Some images were cropped to include only the liver parenchyma and liver masses, while others also contained the surrounding organs. The proposed architecture was used to train five different models that used images from different phases of the CT scanning process: triphasic, arterial, and delayed phase, unenhanced, arterial phase, and delayed phase. In the testing phase, the accuracy of each model in classifying liver masses into five different categories was evaluated. The triphasic and the arterial/delayed models had the best performance compared to the other three. The first model performed the best on the test data, with an accuracy of 84%, and 95% on the training data. The arterial/delayed model performed slightly worse on the test data and slightly better on the training data, but the differences are statistically insignificant. However, the other methods performed significantly worse, thus showing the importance of comparing images at different contrast phases when performing the diagnosis.

Most of the implementations presented above work on segmenting 2D images independently. However, this approach does not take advantage of the contextual information provided by processing multiple sequential slices of the same volume. Abdominal CT scans are usually acquired as 3D images, with slices taken at a known distance, so volumetric information is available, but training a 3D CNN comes with high GPU memory requirements and a longer training time. Xiao Han proposed a 2.5D network architecture, which can take advantage of contextual 3D information without processing the whole volume in one pass [18]. To segment each slice, several adjacent axial slices are selected and processed together. The additional slices only provide contextual information to the network, while the center slice in the stack is the one being segmented. Overall, the architecture is based on U-Net, which was adapted for 3D images to process the image stacks, and to which residual structures were added in both the encoder and decoder. For this implementation, stacks of five adjacent slices were passed through two similar sequential networks. The first provides coarse segmentation of the liver, while the second refines the liver segmentation and discriminates between healthy liver tissue and lesions. The images were preprocessed only by thresholding to eliminate pixels with values outside the liver intensity range. The resulting segmentation was post-processed by thresholding the confidence value of the segmentations to values above 0.8. This implementation achieved a 67% Dice score on the LiTS17 database when considering both liver and tumor segmentation. Another study performed by Wardhana et al. [19] investigated the effects of using different numbers of slices as input, the effects of contrast enhancement techniques, and whether adding an additional encoder–decoder structure brings significant improvements. The slice arrangement study evaluated network performance when trained with one, three, five, seven, or nine slices, and the best performance was achieved when using three slices (one center slice with one adjacent slice on either side). Increasing the number of slices led to a degradation of the network’s performance due to overfitting the training data. The authors decided to use a basic contrast enhancement technique based on value windowing and normalization. Lastly, the study investigates whether any improvements can be made by adding a second encoder–decoder structure to the network, such that the output of the first structure feeds into the second one. The liver segmentation Dice score is similar between the two networks, but the extended network has a lower overall score when it comes to tumor segmentation. As a result of higher sensitivity, the extended network can identify smaller tumors that are not observed by the simple network, while it also misidentifies healthy tissue for tumors and exaggerates tumor size. 

As discussed in the previous paragraphs, segmentation using volumes or stacked slices can prove to be beneficial for liver and tumor segmentation compared to processing individual slices, as it gives contextual information and provides better tissue localization. However, 3D convolutions come with a greater cost of memory consumption and computation time. Besides the 2.5D methods presented above, there are solutions that use different-size networks to process intra-slice and inter-slice features and then combine the results to create a final segmentation. One such method, proposed by Chi et al. [20], uses a multi-branch UNet-like network to create three-dimensional liver and tumor segmentation. The proposed XNet architecture consists of three UNets that share a downsampling and an upsampling path. The main tumor feature extraction network uses a Dense UNet encoder, which provides multi-scale, deep feature analysis using a low number of parameters, which is also shared with the liver mask segmentation network. The decoder path combines shortcut connections from the Dense encoder as well as the filtered liver region network. This XNet structure generates intra-slice liver and tumor feature maps that encode the differences and relations between liver lesions and healthy tissue. To gain inter-slice contextual information, a modified 3D UNet processes the filtered liver volume generated by the previous network. Based on the assumption that the contextual information of a small area in a slice is decided mostly by a similarly sized neighborhood in the adjacent slices, all convolutional layers are sized to 3 × 3 × *n*. Thus, it can detect changes along the z-axis while prohibiting the influence of pixels outside the specified neighborhood. This modification also greatly reduces computation strain as it performs a low number of mathematical operations for each volume. To generate a final segmentation, the intra-slice and inter-slice features are concatenated and then passed through a fully convolutional layer, which reduces the dimensionality of the feature to volumetric lesion segmentation. The system was evaluated on the LiTS dataset, where it achieved state-of-the-art performance.

Based on the results described in the papers above, we chose a set of CNN models suitable for the described application, which will be responsible for segmenting, sequentially, the liver tissue and lesion tissue sequentially.

## 3. Materials and Methods

The proposed method for segmentation involves using multiple classification networks for feature extraction and dedicated segmentation heads to perform segmentation from the resulting feature maps. The model works in a similar way to a UNet, where the image classification network acts as an encoder, and the segmentation head acts as a decoder, reducing the dimensionality of the tensor while upscaling it to the original size. Then, the segmentations obtained from these networks will be used as inputs for a weighted decision system, which will fuse the inputs into a single segmentation. This process is performed separately, first to segment the liver tissue and then to determine the lesion areas, using only the liver as an input. As presented in [21], sequential segmentation approaches yield significantly better results compared to multiclass segmentation in one step.

### 3.1. Image Dataset Used

The reference dataset for liver and liver lesion segmentation from CT images is LiTS17 [7]. This dataset was created in collaboration with seven hospitals and research institutions. Nowadays, the “gold standard” in medical imaging segmentation is a manual segmentation done by expert radiologists, and the authors mention in the article that the LITS17 data was reviewed by three independent radiologists. It includes 131 CT examinations with several types of tumor contrast levels, abnormalities in tissue size, and a varying number of lesions. Another 70 undisclosed volumes are used for model evaluation in the ongoing MICCAI competition. Since the examinations are provided by several different sources, they present a mix of different CT scanners and acquisition protocols. Image quality, resolution, and slice thickness also vary between volumes. Some of the examinations also present imaging artifacts, such as metal artifacts, which can also be present in other clinical data.

### 3.2. Convolutional Neural Networks Used

For these experiments, based on experimental results in similar applications, we chose to use four performant image classification convolutional networks: ResNet, ResNeXt, DenseNet, and Inception. Overall, most image classification networks share the same structure and have multiple similar blocks, which differ only in the values of the hyperparameters. Inside the blocks, the individual layers follow the same structure, which represents the particularity of the network. Each block reduces the size of the input and generates a higher number of feature maps that represent lower-level details. To perform the classification task, the last feature maps are flattened and passed through a structure of fully connected linear layers, which in the end provides a prediction for the specific class to which the image belongs. However, in this application, only the final feature maps are of interest, so only the convolutional part of the networks will be kept, and the output will be passed to the classifier head. The particularities of modifying and training the networks in this manner will be presented in the implementation section.

#### 3.2.1. ResNet152

**Figure 1:**
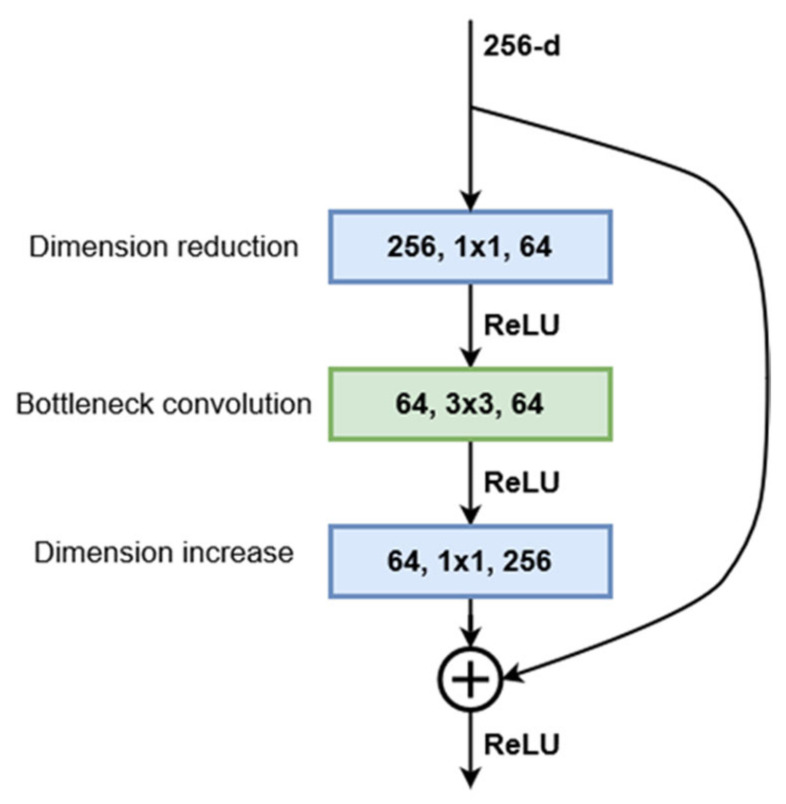
Structure of residual bottleneck building block.

The ResNet architecture was first proposed by He et al. [22]. They showed that this network is easier to optimize and gains considerable accuracy by increasing the depth. The proposed ResNet architecture is based on residual training blocks, which transfer the input of the block and add it to the output without changing it. This feedforward residual path addresses the problem of vanishing gradients and allows the network to benefit from increased depth. Starting with a plain convolutional network with 34 layers, residual connections were added every two layers. This first implementation of ResNet achieved a significant decrease in the top-1 error while having the exact number of parameters as the plain network. To address the higher training times that come with increased network depth, a bottleneck structure was proposed. This consists of 3 convolution layers: 1 × 1, 3 × 3, 1 × 1. The 1 × 1 convolutions are used to first reduce and then restore the dimensions of the input feature maps, leaving the 3 × 3 layer with smaller dimensions (Figure 1). Using this bottleneck structure, similar networks of various depths were designed. The chosen architecture, 152-layer ResNet, is built from 50 sequential bottleneck blocks grouped into four blocks, where each block doubles the number of channels processed by the bottleneck layer. Being designed to be trained and evaluated on the ImageNet dataset, the convolutional sequence of the network is followed by a fully connected linear layer with 1000 outputs, which represent the predictions for the 1000 classes in the dataset. However, since we used this network as a feature extractor, the output of the last convolutional layer will be transferred to the DeepLab module. In the case of ResNet152, this output consists of 2048 8 × 8 feature maps for inputs of size 256 × 256. Figure 1 presents the structure of the bottleneck block used in the current ResNet implementation.

The ResNet block can be described mathematically by Equation (1), which can also be used to compute the block parameter gradients during model training, where *W_i_* are the convolution weights and *B_i_* are the convolution biases if they exist:

(1) $$Y=ReLU\left(X+\left(W_{3}*ReLU\left(W_{2}*ReLU\left(W_{1}*X+B_{1}\right)+B_{2}\right)+B_{3}\right)\right)$$

#### 3.2.2. ResNeXt101

**Figure 2:**
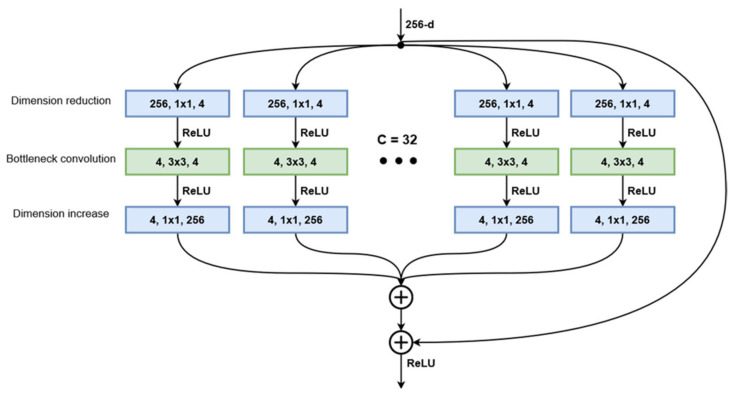
Structure of the split-transform aggregate block with cardinality 32 and bottleneck width 4.

Starting with the standard ResNet structure, the authors of [23] developed a modified version of this architecture that implements aggregated transformations. The general design follows the modularity of ResNets, stacking residual blocks with the same topology. Blocks processing the same number of feature maps have the same width and filter size, and when the spatial map is downsampled by a factor of 2, the block width is multiplied by the same factor. Taking inspiration from Inception networks, the ResNeXt blocks implement the split-transform-aggregate strategy. The bottleneck structure from the original ResNet is split into multiple paths where the 3 × 3 convolution layer operates on a significantly smaller number of channels (Figure 2). The number of distinct paths in a block is referred to as cardinality and is considered an additional hyperparameter when creating the network structure. At the end of the block, the outputs of all paths are summed together, and the block input is added via the residual connection. Their experiments showed that while maintaining the same number of parameters, a ResNeXt50 network, with a cardinality of 32 and bottleneck width 4, outperforms a ResNet50 model with bottleneck width 64, having a top-1 error 1.7 percentage points lower. Further examinations showed that increasing the cardinality of the aggregated transformation structures produces much better results than increasing the network depth or widening the bottleneck layer. The ResNeXt101 variant, which will be used, is built from 33 stacked bottleneck modules grouped in four blocks that follow the same rule for increasing the number of channels as the ResNet variant presented above. Similarly, the convolutional layers end with a fully connected layer that computes the activation values for the 1000 classes of the ImageNet dataset. This layer is of no interest for the implementation since we will be using the network as a feature extractor, not a classifier.

The equation that describes the ResNeXt block output in relation to its input (2) is similar to the ResNet block but takes into account the different weights and biases across the parallel convolutional paths.

(2) $$Y=ReLU\left(X+\overset{32}{\underset{i=1}{\sum }}\left(W_{i,\;3}*ReLU\left(W_{i,2}*ReLU\left(W_{i,1}*X+B_{i,1}\right)+B_{i,2}\right)+B_{i,3}\right)\right)$$

#### 3.2.3. DenseNet201

**Figure 3:**
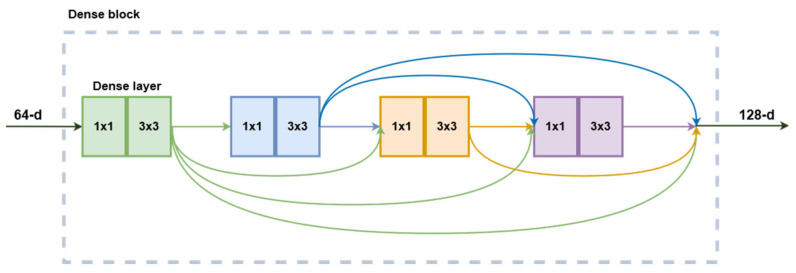
DenseNet building block with 4 dense layers. Each rectangle corresponds to a sequence of BatchNorm-ReLU-Convolution.

Much like the other networks presented above, the DenseNet structure was designed to perform as an image classifier network. As described in [24], this architecture continues the philosophy of stacking convolutional layers with the same structure and different hyperparameters. However, instead of relying on network depth or width for increased performance, this family of CNNs exploits the potential for feature reuse. Building on the ResNet concept of feedforwarding the layer input its output, this implementation introduces direct connections from every layer to all subsequent layers in a feedforward fashion. In this case, the feedforward connection is made by concatenation instead of summation such that each layer receives the output feature maps from all the layers before it. The composition function of a dense layer is composed of six consecutive operations: batch normalization, ReLU activation, 1 × 1 convolution, batch normalization, ReLU activation, and a 3 × 3 convolution. The first three modules function as bottlenecks, while the next three are responsible for actual image processing. Multiple layers are stacked in a dense block and share the same hyperparameters. The dense feedforward connections only happen inside the blocks, not between them since the size of the feature maps changes. Between blocks, a transitional layer composed of a batch normalization, 1 × 1 convolution followed by 2 × 2 average pooling is responsible for reducing the dimensions of the feature maps for the next block. The DenseNet201 variant is built from four dense blocks (Figure 3), with a total of 98 dense layers. Their tests showed that DenseNets are more efficient in terms of parameters than ResNets. In terms of prediction accuracy on ImageNet, DenseNet201 is equivalent to ResNet101 while having less than half the number of trainable parameters. 

For this block’s mathematical representation, let *F_i_* be the activation of dense layer *i*, and Z*_i_* their respective outputs. Then, we can write the following:

(3) $$F_{i}\left(X\right)=\left(W_{i,2}*ReLU\left(W_{i,1}*ReLU\left(X\right)+B_{i,1}\right)+B_{i,2}\right)$$

(4) $$Z_{1}=F_{1}\left(X\right)$$

(5) $$Z_{2}=F_{2}\left(Z_{1}\right)$$

(6) $$Z_{3}=F_{3}\left(\left[Z_{1},\;Z_{2}\right]\right)$$

(7) $$Z_{4}=F_{4}\left(\left[Z_{1},Z_{2},Z_{3}\right]\right)$$

(8) $$Y=\left[Z_{1},Z_{2},\;Z_{3},Z_{4}\right]$$

Equations (3)–(7) can be further substituted into (8) to expand the representation of the final layer’s output.

#### 3.2.4. InceptionV3 

**Figure 4:**
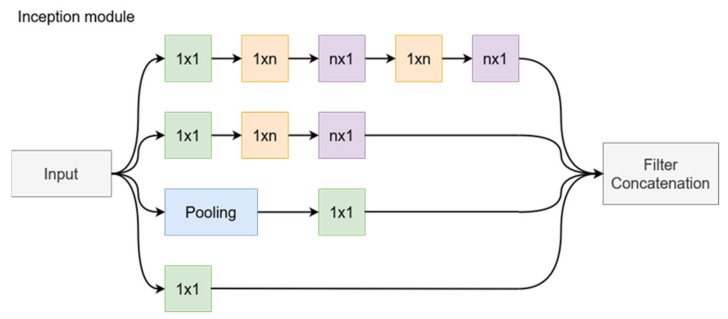
The general structure of an inception module after the factorization of an *n* × *n* filter.

The creation of the Inception architecture, as presented in [25], had the objective of scaling up networks in such a way that the added computational complexity could be used as efficiently as possible. The computational cost of Inception networks is significantly lower than that of VGGNet or its successors with higher performance; however, its structural complexity makes it more difficult to make changes to the network. For example, trying to increase the network capacity through doubling the filter bank size will lead to a four times increase in computational cost and network parameters, but the performance increase might be insignificant. This paper presents some of the design principles that went into the design of Inception networks. First, the design follows the principle of avoiding bottlenecks, especially in the initial stages of the network. It reduces the dimensions step by step when going deeper into the network. In addition, higher dimensional representations are easier to process locally, and network training is faster when the activations per tile are increased. Regarding network hyperparameters, the Inception design balances the width and depth of the network, increasing them in parallel to achieve optimal improvements. The original Inception architecture [26] is built from multiple Inception blocks. Each block sends its inputs along four parallel paths, and the outputs of each path are concatenated at the end of the block (Figure 4). In the naïve implementation, these paths contain the following operations: 1 × 1 convolution, 3 × 3 convolution, 5 × 5 convolution, and a 3 × 3 max pooling. The reasoning is that instead of choosing a single type of operation, the network could train the parameters for each operation as needed at various depths. However, this approach leads to immense computational costs. To address this issue, the original network adds a 1 × 1 convolution before the 3 × 3 and 5 × 5 convolutions to function as a bottleneck layer, thus leaving fewer dimensions for the complex convolutional layers. The new Inception design goes one step further to improve the efficiency by replacing the 5 × 5 convolution with two sequential 3 × 3 convolutions. Since the 5 × 5 convolution is 2.78 times more computationally expensive than the 3 × 3 variant, this approach proves to be more effective. Efficiency can be increased even further by employing spatial factorization into asymmetric convolutions. This way, a 3 × 3 convolution is split in a 3 × 1 followed by a 1 × 3 convolution. This two-layer solution is 33% cheaper in terms of computational complexity. This method was shown to not work well in the early layers but is effective when used for the factorization of *n* × *n* grids with *n* between 12 and 20. Figure 4 presents the general structure of an Inception block with filter size *n* × *n*. The last implemented change is to use the two asymmetrical convolutions in parallel, thus expanding the size of the filter bank. These changes are implemented gradually in the network. To reduce the grid size between Inception block groups, the last block of the group implements a stride of 2 in all parallel operations, thus halving the width and height of the feature maps. 

As for the previously presented model, to describe its behavior mathematically, we will first the output of each path as a function of the input tensor and then concatenate these outputs to get the final block output, as in Equation (13).

(9) $$Z_{1}=W_{1}*X+B_{1}$$

(10) $$Z_{2}=W_{2}\left(MaxPool\left(X\right)\right)+B_{2}$$

(11) $$Z_{3}=W_{5}*\left(W_{4}*(W_{3}*X+B_{3}\right)+B_{4})+B_{5}$$

(12) $$Z_{4}=W_{10}*\left(W_{9}*\left(W_{8}*\left(W_{7}*\left(W_{6}*X+B_{6}\right)+B_{7}\right)+B_{8}\right)+B_{9}\right)+B_{10}$$

(13) $$Y=\left[Z_{1},Z_{2},Z_{3},Z_{4}\right]$$

#### 3.2.5. Segmentation Head

**Figure 5:**
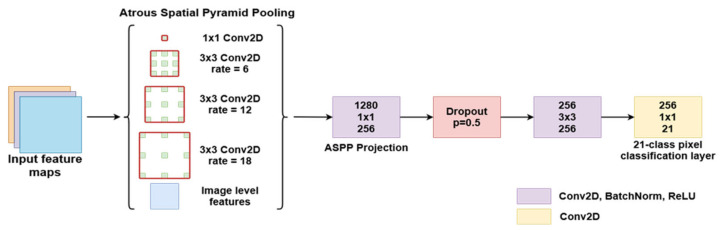
DeepLabV3 segmentation structure.

Atrous convolution is a powerful method that can be used to adjust a filter’s field of view as well as to accurately control the resolution of features computed by a CNN. The authors in [27] presented a solution for solving semantic segmentation at multiple scales by using parallel and cascade atrous convolution modules and image-level features to further boost performance. The DeepLabV3 network structure (Figure 5) expands on the concepts of atrous convolution, which allows the network to train feature detection at different scales without downsampling the image. Modern Deep Convolutional Networks used for segmentation or classification usually decrease the input image size by a factor of 32 through multiple max pooling and striding layers. Transposed convolutions are usually used to restore the image to the original size. The motivation behind using atrous convolutions is that the image only needs to be decimated to a lesser extent, which allows for control of the feature resolutions through the kernel dilation rate. This technique relies on upsampling convolution kernels at different rates by spacing out the kernel and inserting zeros between consecutive kernel values.

The experiments started with a standard ResNet structure. The last block was repeated 4 times and added in sequence to the network, each time increasing the dilation of the atrous convolution kernel. To allow all the different resolution filters to work on unaltered data, additional filters were added in a parallel manner using the ASPP concept. This structure produces features at multiple resolutions and concatenates them for the final segmentation layers. This method allows the network to achieve multiple effective field-of-views, which leads to the ability to encode multi-scale feature information. However, the dilation rate of the kernel should be carefully chosen in relation to the size of the feature maps. If the dilated extremities of the kernel fall outside the valid image space, then the convolution effectively becomes a 1 × 1 filter, which is not desirable. Along with the multi-resolution feature maps, image-level features are added by resampling the input at the expected output size. This semantic segmentation network was trained and tested with a ResNet50 and ResNet101 backbone and performed better with other state-of-the-art models on the PASCAL VOC 2012 dataset, achieving an 86.9% mIoU score. However, this segmentation method could be adapted to work with any image classification network with minor adjustments, effectively transforming it into a semantic segmentation network.

The actual semantic segmentation structure passes the input through four parallel paths, each with a convolutional layer employing a different atrous rate (Figure 5). The outputs are concatenated with the image-level features and then passed through another two Conv-BatchNorm-ReLU blocks. The first block reduces the number of feature maps through a 1 × 1 convolution and is followed by a Dropout layer with 0.5 probability, which randomly zeroes some of the elements of the input tensor, based on a Bernoulli distribution. The use of Dropout layers proved to be an effective tool for network regularization during training and prevented neuron co-adaptation, as described in [28]. At the end, a 1×1 filter reduces the number of feature maps from 256 to the number of classes, providing per-pixel classification. If necessary, the image is bilinearly upsampled back to its original size.

This structure will be appended to the image classification networks described above to generate liver and lesion segmentations from the provided feature maps.

### 3.3. Decision Fusion System

The proposed system, as described until now, produces four different segmentations for one input image passed through the four networks described above. These results will be fused into a single segmentation that should have improved performance metrics. To achieve this, we proposed a weighted average system in which the weights could be individually adjusted for each network. The reasoning behind this approach is that each different network will learn different low-level features, such that segmentation errors will appear in different places in the image for each network result. Applying a weighted average over these results may result in errors from each network being corrected by the other three networks. 

**Figure 6:**
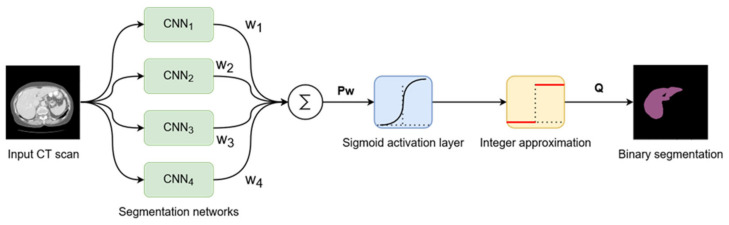
Modular representation of the decision fusion system.

The input image is passed as input to each segmentation network, and the results are computed. Then, each segmentation is multiplied by its corresponding weight, and they are all added together. At the end, the result is passed through a Sigmoid activation function, and the values are rounded to the nearest integer to provide a binary segmentation mask (Figure 6).

Suppose that *P*_1_ to *P*_4_ are the segmentations provided by the four segmentation networks, and *w*_1_ to *w*_4_ their corresponding weights. Then, the fusion system’s output *Q* can be described as follows (14):

(14) $$\begin{array}{c} P=\left[P_{1},P_{2},P_{3},P_{4}\right]\\ w={\left[w_{1},w_{2},w_{3},w_{4}\right]}^{T}\\ \sigma \left(x\right)=\frac{1}{1+e^{-x}}\\ Q=round\left(\sigma \left(Pw\right)\right) \end{array}$$

The trivial choice of weights would be to give them the same value. However, since network performance is not the same across all networks, weighting based on performance could be a better idea, since better-performing networks would have a higher influence on the final segmentation. Our choice is to programmatically adjust the weights individually to minimize a cost function in the same way that linear neural networks are trained. The criterion used in the proposed method is binary cross entropy, so the following cost function to minimize is obtained (15), where *G* is the ground truth segmentation mask:

(15) $$\underset{w\in {\mathbb{R}}^{4}}{min}\left|\frac{1}{N}\sum \left(Glog\left(\sigma \left(Pw\right)\right)+\left(1-G\right)log\left(1-\sigma \left(Pw\right)\right)\right)\right|$$

The set of weights that minimizes the cost function can be found using iterative gradient descent algorithms, such as Stochastic Gradient Descent. The details of weight adjustment after the networks are trained will be presented in the Implementation section.

### 3.4. Segmentation Performance Metrics

In the domain of biomedical image segmentation, the most widely used metrics to assess performance are the Dice Similarity Coefficient (*DSC*) and the Jaccard index. In this application, we evaluated the *DSC* on binary segmentation masks separately for liver segmentation and lesion segmentation. This metric measures the degree of overlap between the ground truth and the evaluated system segmentation. Given two binary masks, *A* and *B*, *DSC* is evaluated using the following formula (16):

(16) $$DSC\left(A,B\right)=\frac{2\left|A\cap B\right|}{\left|A\right|+\left|B\right|}$$

This formula produces a value in the range [0, 1], where 1 signifies perfect overlap. False positives, as well as false negatives, have a negative impact on the Dice metric. The result depends, however, on the size of the erroneous region compared to the cumulative size of the reference and evaluated segmentations. This means that missing a small lesion in an image that has multiple larger lesions will have a low impact on the score, while missing a small lesion in an image that presents only that lesion will produce a score of 0. To address this variation based on the total segmentation mask area, two metrics are usually computed. The Global Dice coefficient is evaluated for all examinations combined into a single volume and is more sensitive to larger lesions. The Dice coefficient per case is computed for each individual volume, then averaged over all examinations, and a higher penalty is applied to volumes with smaller lesions.

## 4. System Implementation

### 4.1. Deep Learning Frameworks

The programming language used for this implementation is Python 3.8. We chose this language because it is very flexible for prototyping, has a very good ecosystem for scientific computing through SciPy, provides Matlab-like syntax for matrix and vector operations with NumPy, and provides an interface for traditional machine learning methods through scikit-learn. Additionally, to process the initial CT image database, which is in the Neuroimaging Informatics Technology Initiative (NifTI) format, the NiBabel library [29] was employed to extract the raw data from the volumetric examinations. To perform image pre-processing and post-processing, we used scikit-image, a community-developed library that provides easy access to a vast collection of image-processing algorithms [30]. Since not all medical images come in the same format, even though the database was stored in the NifTI format, other databases, such as 3Dircad and local hospital resources, are stored in the DICOM format, so we used the pydicom library [31] to extract the pixel values, modify metadata, and add colored ROIs on the segmented examinations. To create all visualizations related to the project, we used Matplotlib, a Python library, to create static and interactive visualizations, from statistics to images [32].

The two most popular frameworks for Python development are PyTorch, developed by Facebook, and TensorFlow, developed by Google. We chose to use PyTorch [33] because it has several qualities that make it preferable to prototype new architectures. First, it supports code as a model design principle, which enables easier modification of existing models and creation of new models with minimal effort, since network layers are viewed as classes that process some input through a forward function and provide a built-in differentiation mechanism. This design model is further facilitated using a dynamic computation graph, which provides extended flexibility and the option of modifying model structures at runtime, as opposed to TensorFlow, which uses static computation graphs until later versions. While TensorFlow requires a separate debugger to examine the data flow inside models, PyTorch allows the standard Python debugger to break execution and examine variables inside models during runtime. 

In addition to these advantages, this framework presents a comprehensive list of features that facilitate the fast development of deep learning workflows. Similar to most other deep learning frameworks, PyTorch is built upon a reverse-mode automatic differentiation algorithm, which lets it automatically compute gradients of custom models, or even for common programs that perform mathematical calculations and employ gradient-based optimizations. The differentiation mechanism is based on operator overloading, which stores the mathematical function every time a supported function is applied to a tensor that tracks gradients. To aid with workflow development, PyTorch implements data loader classes tailored for image classification, which automatically generate labels and split the dataset. Moreover, reference models, such as AlexNet, VGGNets, and even newer models, such as ConvNeXt and Vision Transformers, are available to train, evaluate, and modify using the torchvision module. It also provides implementations of various cost functions, such as cross-entropy loss, MSE loss, and L1 norm loss. To perform the actual network training, a series of optimizers can be used, starting with the well-known Stochastic Gradient Descent with momentum, and offering a multitude of other algorithms, such as Adam, Adagrad, RMSprop, Adadelta, and LBFGS. To accelerate deep-learning routines, this framework provides easy training using GPUs, thanks to its C++ core, which interfaces with CUDA runtime, thus providing low-level, fast access to graphics computational resources.

### 4.2. CT Images Pre-Processing

The CT images in the LiTS17 dataset are stored in NIfTI format, with each file representing an entire examination volume. Of the 131 examinations, 121 were used for network training and the remaining 10 for testing and determining the Dice scores. To make data loading easier, the volumes were split into individual slices. In this step, a windowing function is applied, which clamps the image values between −250 and 250 HU. Then, the pixel values are rescaled to the interval [0, 1]. This ensures that there won’t be major contrast differences between the images during testing. To reduce the file size, all metadata were eliminated, and the slices are stored as raw pixel values. In this way, individual slices can be randomly loaded without loading the entire volume. The initial image size is 512 × 512 pixels. To reduce the training time and memory requirements, we downsampled both the images and ground truth segmentation masks used for training to 256 × 256 pixels. Of course, training on downsampled images will reduce segmentation accuracy, since the evaluation will be done on full-resolution examinations, but it is a trade-off between performance, training time, and the practicality to train on available hardware. Furthermore, the networks used in this implementation contain Batch Normalization layers for feature map regularization, and these layers learn the statistical properties of the training dataset. By applying the mentioned downsampling, the training batch size can be increased by a factor of 4, assuring that the BatchNorm layers will learn more statistically significant metrics provided by a larger sample size. For the testing dataset, however, only the images were downsampled, which ensured a fair evaluation of the Dice scores, which would not be possible if the ground truth masks were also downsampled.

### 4.3. Segmented Liver Image Processing

As discussed, the segmentation system operates in two distinct stages, such that lesion segmentation is performed on images that contain only the liver, without any surrounding tissue. To perform liver extraction and to prepare the images for training, a series of steps are executed, some of which are similar to CT pre-processing. First, we applied the same windowing function, which limits the intensity values to −250 to 250 HU. Then, the values are shifted by 250 and divided by 500 to occupy the range [0, 1]. The next step is to apply liver segmentation, which was performed in the previous stage, as a transparent mask, which is done by multiplying the individual CT image pixels with the mask pixels. This will retain the original values inside the hepatic region while setting the surrounding tissue and background to a value of 0. Then, the x, y, and z direction extremities of the liver volume need to be determined to generate a volume of interest. This is done by iterating through the slices from the three axes and retaining the minimum and maximum slice indexes, where there is a non-zero value. The volume of interest is cropped from the original CT examination, thus eliminating any unnecessary regions and all slices that did not contain liver tissue. Then, each slice is resized to 256 × 256 pixels, such that all images have the same size during training. This method relies on the previous segmentation being correct. 

**Figure 7:**
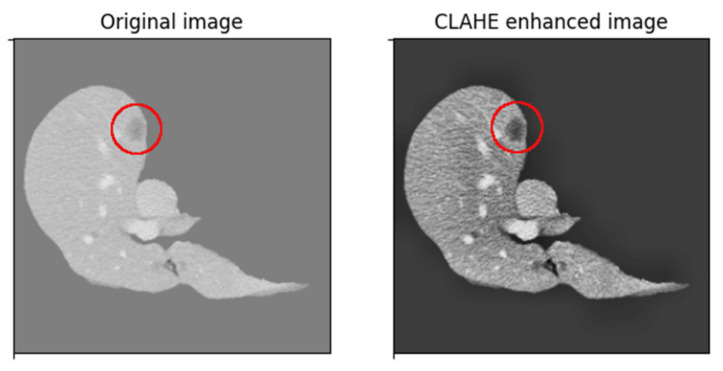
CLAHE increases lesion contrast.

Since liver lesions have a similar intensity to the surrounding tissue, a contrast-enhancing method is used to create better differentiation. First, we passed through each volume slice and assigned the background pixels, which had a value of zero and a value equal to the mean of nonzero pixels in the slice. This both limits the image’s initial dynamic range and eliminates strong edges around the liver, which have a strong effect on network activation but are not needed for lesion segmentation. The method used in this implementation is based on Contrast Limited Adaptive Histogram Equalization (CLAHE). CLAHE aims to improve some of the drawbacks by dividing the image into 8 × 8 blocks, then performing histogram equalization on each block individually, which incorporates contextual information and improves contrast, at the cost of amplifying noise in constant regions. CLAHE limits contrast enhancement, specifically in homogeneous regions where the histogram presents very high and narrow peaks. In these regions, the slope associated with the gray-level redistribution function is reduced, limiting noise amplification. The result of this histogram equalization method can be seen in the image below (Figure 7), where the lesion area becomes more visible.

### 4.4. From Image Classification to Semantic Segmentation 

As discussed in the previous sections, an important contribution of this project is to study how image classification networks can be used as feature extractors to perform semantic segmentation and to implement a solution based on this methodology. This section will provide detailed descriptions of the structural changes made to the networks, and how the data flow was modified to fit the task we want to achieve. The general rule is that since classification networks are composed of a convolutional stage that performs feature extraction and a linear stage that performs classification, the latter should be removed, and instead a segmentation head, such as FCN or DeepLab, can be connected.

#### 4.4.1. ResNet152

The software implementation of this ResNet variant is composed of multiple grouped layers. First, there is a 7 × 7 convolutional layer, which takes images with 3 channels and outputs a tensor with 64 feature maps. This layer was changed with one of the same kernel sizes, but it accepts single-channel images because the medical examinations are grayscale. This is followed by a sequence of 4 blocks that contain 3, 8, 36, and 3 bottleneck blocks, which share the same hyperparameters inside the blocks. The tensor dimensions are reduced immediately after the first convolutional layer through a Max Pooling layer. Each bottleneck block contains 3 individual convolutional layers; thus, the 152 layers that characterize this network are obtained. This convolutional part is followed by global average pooling and a fully connected layer, but these are eliminated because they are only necessary for classification. The output is taken directly from the fourth structural block, which has a size of (b, 32, 32, 2048), where b is the batch size. This means that each 256 × 256 input image was transformed into 2048 32 × 32 feature maps. In the modified network architecture, this feature map block is passed to the DeepLab segmentation head. In the original DeepLabV3 implementation, the kernel dilation rates were set to 12, 24, and 36 because they were trained on significantly larger images of the COCO dataset. For feature maps of size 32 × 32, both kernels with dilation 24 and 36 would act as 1 × 1 convolutions since the outer kernel elements will be outside the image bounds. Therefore, the dilation rates were reduced to 4, 8, and 12. In addition, the last convolutional layer of the segmentation dataset was replaced, such that it outputs an image with only one channel, which represents the pixel probabilities of containing liver or lesion tissue, depending on the training case.

#### 4.4.2. ResNeXt101

The software implementation of this ResNeXt variant is composed of multiple grouped layers, similar to the network described above. Similar to its ResNet counterpart, there is a 7 × 7 convolutional layer, which takes images with three channels and outputs a tensor with 64 feature maps. This layer was changed with one of the same kernel sizes, but which accepts single-channel images, since the medical examinations are grayscale. This is followed by a sequence of four blocks that contain 3, 4, 23, and 3 bottleneck blocks, which share the same hyperparameters inside the blocks. However, since this network implementation uses grouped convolution for the bottleneck blocks, we also must specify its variant, which in this case is 32 × 8 d. This means that inside each bottleneck layer, a set of 32 grouped convolutions is computed, each having an input and output size of 8 × 8 pixels. This convolutional part is followed by a global average pooling and a fully connected layer, but these are eliminated since they are only necessary for classification, as in the previous case. The output of the fourth structural block has the same shape as that of the previous network, so we made the same modifications based on the same reasoning for this network.

#### 4.4.3. DenseNet201

The software implementation of this DenseNet variant is composed of multiple structural blocks: dense blocks and transition blocks. First, there is a 7 × 7 convolutional layer, which takes images with three channels and outputs a tensor with 64 feature maps. This layer was changed with one of the same kernel sizes, but it accepted single channel images. It is followed by a sequence of four dense blocks composed of 6, 12, 48, and 32 sequences of BatchNorm-ReLU-Conv2D-BatchNorm-ReLU-Conv2D. These sequences are densely connected inside the structural blocks, but no dense connections appeared between the dense blocks. Between every two dense blocks, there is a transition block, which performs a convolution and an average pooling with kernel size 2 and stride 2, effectively reducing the in-plane feature map dimensions by a factor of 2 along each axis. As before, the linear classification part of the network was removed. In the original formulation of the network, the tensor shape of the last convolutional layer for an input of size 256 × 256 was (b, 8, 8, 1920). This tensor has in-plane dimensions that are too small to generate a satisfactory segmentation result when they are upscaled to 256 × 256 by the segmentation head. To address this issue, we modified the transition blocks such that only the first one performs average pooling, while in the second and third it is eliminated. This increases the output tensor in-plane dimension to 32 × 32, identical to the ResNet variants, but with a different number of channels. The next steps are repeated in the same manner as for the previous networks.

#### 4.4.4. InceptionV3

The software implementation of the Inception architecture is composed of a convolutional sequence and an inception sequence. First, there is a 3 × 3 convolutional layer, which takes images with three channels and outputs a tensor with 32 feature maps. This layer was changed with one of the same kernel sizes, but it accepted single channel images. This is the first in a group of five convolutional layers, which also contains max pooling layers after the third and fifth convolutional layers. This sequence outputs a tensor of size (b, 35, 35, 192), which is then passed to the Inception sequence. This is composed of three different Inception designs, each with 3, 5, and 2 modules. Each design implements successively more complex and deeper asymmetric convolutions. After this sequence, there is the classification part of the network, which we eliminated to extract only the feature maps, which have a size of (b, 8, 8, 2048). As in the case presented above, the small in-plane dimensions make this a less-than-ideal case for upsampling when generating segmentation masks. The issue was addressed by removing the max pooling layers from the convolutional sequence, thus increasing the in-plane dimensions to 32 × 32. The steps to connect the segmentation head to the feature extraction output are the same as in the other three models. The Inception model implementation also provides an auxiliary output, which taps into the intermediate output of the network, after 8 Inception blocks. This is generally used to improve convergence during training by combating the vanishing gradient problem. However, it is not used in this case, as it would require an additional DeepLab module, which would greatly increase training time and memory costs.

### 4.5. PyTorch Dataset and Dataloader

In the PyTorch framework, data structured for training are loaded using extensions of the Dataset class. This provides an interface between the routines that read the data from the disk and perform transformations and the routines that arrange data into randomized training batches, with their corresponding labels or masks. PyTorch offers pre-built Dataset classes for the most common image datasets, such as CIFAR, COCO, Cityscapes, and some video datasets, such as Kinetics and HMDB51. It also provides a template dataset for creating a custom image dataset where classification labels are generated based on the folder name in which the images are in. Since there is no implementation for the LiTS17 dataset, we had to implement my own solution. To do this, we first created a class that inherits the base Dataset class. It takes as parameters a root directory for the images and mask folders, a transformation for the image, and one for the mask. These transformations can be used to resize the images according to specific needs, adjust contrast, and introduce data augmentations in the case of smaller datasets. When the lass is initialized, it generates a list of all files found in the images and masks sub-directories of the root directory and sorts them alphabetically. Then, when a certain image index is requested, the class looks up the corresponding image and mask path and loads them into NumPy arrays, interpreting them as binary files. Then, knowing the pixel count, the arrays are reshaped into 2D matrices to which the specified transforms are applied, if any. The output of this loading routine is a pair of PyTorch tensors, one representing the image data and one representing the mask data. By default, the only transformation I use is image normalization, with a mean of 0.5 and a standard deviation of 0.5. This will move the pixel values into the range [−1, 1]. No resizing is done at this point, as it was performed in the pre-processing stage of the original dataset. The routines implemented only handle loading the data from the disk and preparing it, while another DataLoader class generates training batches. For each epoch, it randomizes the order of the image indices in the dataset. Then, it sequentially samples a block of 32 images and concatenates them to generate training mini-batches. This step of mini-batch randomization is essential for successfully training Batch Normalization layers since successively processing a random subset of samples will give better statistics on the global dataset.

### 4.6. Liver and Lesion Segmentation Post-Processing 

**Figure 8:**
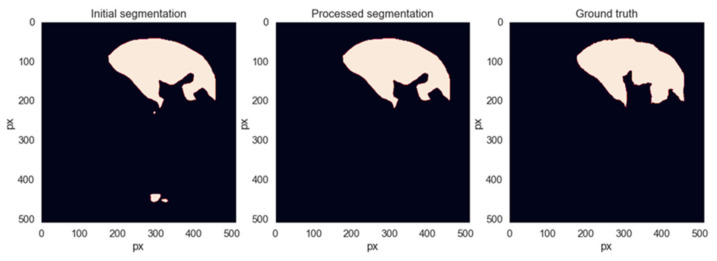
Example of segmentation post-processing.

After the segmentations are generated by the networks, a sequence of additional processing operations will be applied to them to remove segmentation artifacts that do not belong to the liver area. Each slice is a binary segmentation mask and is processed individually. The first step is to find all connected regions and label them individually. If the number of regions is less than 2, the image is left as is. Otherwise, the centroid and area metrics are computed for each connected region and sorted descending by area. The region with the biggest area is the main liver area. Then, we iterate through all the remaining regions and remove them if the distance to the main area is greater than 200 pixels or if its area is less than 8 times smaller than that of the main liver region. These decision thresholds were found empirically, trying to eliminate as many erroneous segmentations from the spleen or intestine area without removing any correct liver segmentations. Figure 8 shows an example of when this method removes unwanted regions from the segmentation while leaving the liver region intact.

To refine the lesion segmentation masks, the same method used for liver masks cannot be employed since these lesions can appear anywhere inside the liver tissue area and can have various sizes, independent of one another. However, the masks will be processed by setting a minimum area for a tumor to be registered; otherwise, they will be deleted. The threshold chosen for this operation is 10 pixels. This value was determined empirically, as it eliminates false positives caused by image granularity and noise.

### 4.7. Creating the Final Segmentation

The two fusion systems that were trained each output a segmentation mask for each input image, one for the liver tissue and one for the lesion tissue, each mask having a size of 256 × 256 pixels. The liver tissue mask can easily be upscaled using bilinear interpolation to match the input image size and will have a direct overlap with the liver tissue in the initial CT image. However, the same steps cannot be applied to the lesion mask, as it was cropped via the determined volume of interest from the volumetric representation of the examination. To be able to overlap the mask on the initial image, the same volume of Interest (VOI) must be determined using the same method described before. Then, we resize the 256 × 256 segmentation mask to match the in-plane dimensions of the VOI. Considering the position and dimensions of the VOI, the resized mask is padded with zeros in all directions to match the 512 × 512 dimension of the initial examination. Moreover, empty masks need to be added before and after the lesion masks to account for the examination slices that do not contain liver tissue and were not processed in the second segmentation step. By following these steps, two maps are obtained that match the dimensions of the CT examination, so they can be used either as semi-transparent colored masks to display the result in a way that is easier to understand for a human operator or can be used to compute additional metrics that could be helpful for diagnostics, such as liver volume or lesion volume, also referred to as tumor burden.

### 4.8. Adaptation to Medical Applications

Having a model that provides good segmentation metrics is essential for a functional biomedical diagnostics system, but it also must be functional in a real scenario, such as a clinical environment, not only in the development environment, where it processes well-structured data with a known standardized format, and its performances are analyzed internally against a reference ground truth. Therefore, a solution is developed that can load DICOM examinations and generate the corresponding result in the DICOM format, such that they can be easily loaded into specialized programs that are available on computers in medical institutions. These programs provide an extensive array of measurement tools that can aid in deciding on a diagnostic, so saving the results in this standard medical format is essential. Usually, CT examinations saved in DICOM format are stored as individual slices, where all slices of an examination are stored in the same folder and the alphabetical order of files defines their order in the CT sequence. These files contain a great number of metadata that define their order in volume, pixel size, slice thickness, patient data, CT acquisition protocol, and X-ray energy, among others. These fields are organized by DICOM tags.

First, the application loads each file listed in the selected directory using a dedicated Python library. Then, the raw pixel values are extracted and processed using a modality lookup table specified by the DICOM header. The data structures are transformed into NumPy arrays and are then processed in the same way as the LiTS dataset: pre-processing, two-stage segmentation, post-processing, and mask resizing and padding. 

To generate the output DICOM, the initial object is copied with all metadata. The original CT image is converted from grayscale to RGB by repeating the gray channel 2 times. This allows for colors to be added to the image. The liver mask is given a green color and an alpha value of 0.3, and is overlaid on the initial examination. The lesion mask has the same transparency value but a red color and is overlaid in the same manner. However, for DICOM viewers to be able to register these changes, a few modifications to the metadata need to be made. First, the PhotometricInterpretation tag must be set to RGB, and the SamplesPerPixel to 3, since each pixel is now represented by 3 bytes, one for each color channel. Next, the HighBit tag is set to 7 to specify that the data are stored in the little-endian format. The series number is incremented, so the file viewer will see them as 2 different datasets, then the files can be saved to disk. The result is that the segmentation results can be viewed interactively as colored regions overlaid on the initial CT examination.

## 5. Experimental Results and Discussion

This section will present the experimental results, which are the outcomes of the work presented in this paper. The metric used is the Dice similarity index, as this allows comparisons to be made to other solutions trained on the same dataset. The system’s performance was evaluated on the testing dataset, ensuring that none of the images were seen during the training phase. Even though the system processes images of a smaller size, the segmentations were upsampled to match the ground truth size provided in the dataset to create a fair comparison.

### 5.1. System Training

#### 5.1.1. Training Segmentation Networks

The training of the models that made up the proposed system was done in a sequential manner: first, we trained liver segmentation variants, then the lesion segmentation variants of the same models. However, training in this order is not compulsory, since lesion training was done with the reference segmentations, and not with the segmentation provided by the previous step. All four segmentation models were trained individually. The feature extractor part of every network uses pre-trained weights from the PyTorch database. These are the weights corresponding to models trained on the 1000-class ImageNet dataset. This means that the models can already extract features relevant for classification, which should reduce the training time for this new task. The weights and biases used for the segmentation head of each model are initialized using the Kaiming method. Even if the feature extraction section uses pre-trained weights, these weights are also optimized by the training algorithm. Since the first convolutional layer of each network was replaced to accommodate grayscale images, it has random weights and should be optimized, so its gradients must be computed. Since this can only be done by computing the gradients of all downstream layers, all layers can be optimized with a negligible increase in execution time. Most of the computation time is dedicated to network inference and gradient backpropagation, not to weight adjustment via gradient-based methods.

Each liver segmentation model was trained for 200 epochs using a stochastic gradient descent (SGD) optimizer. We set the learning rate to 0.05 and the momentum to 0.9. As shown by [33], using momentum and choosing a suitable model initialization can lead to better performance and faster convergence, as it accelerates gradient descent in the direction of a persistent decrease in the cost function. To aid with convergence, as the model parameters approach a point of minimum, a learning rate scheduler is used, which decreases the learning rate every 10 epochs by 5%. In this way, the step size of the gradient descent optimizer becomes smaller as it approaches a solution, so it cannot “jump” to a nearby local minimum. This also decreases overshoot when searching for the optimum point, so it can converge in fewer steps. The training was conducted in batches of 32 images, which was the largest size that could be loaded onto the available hardware. 

For the lesion segmentation models, we used the network parameters learned in the previous step as a starting point for model training. The reasoning behind this is that previous networks learned to distinguish between liver features and other organs, so this information could be transferred faster to particularities of liver tissue, such as lesions. Since we are starting with models that can already perform liver localization, a higher learning rate of 0.1 was set, and a more aggressive learning rate scheduler, where the learning rate is halved after each epoch. This ensures that the model parameters will move faster toward the optimum at the beginning of the optimization, and then the later epochs will perform a very fine parameter tuning to move as close as possible to the minimum point. The training is done for only 20 epochs, as further iteration caused the learning rate to become too small to make effective improvements. The learning rate for the last epoch is 1.9 × 10^−7^. These values for learning rate decay, initial learning rate, and total number of epochs were determined empirically after running multiple tests and comparing the evolution of the loss function. More tests with various training hyper-parameter value combinations may result in a better starting point and schedule, but we were limited by computation time restrictions. 

The machine used for training is configured as a SLURM partition, where jobs can be submitted by individual users. It is equipped with two 20-core Intel Xeon E5-2670 CPU, 64 GB of DDR3 RAM, and three NVIDIA Tesla K40m GPUs with 12 GB of GDDR5 VRAM, each processing unit having 2880 cores.

#### 5.1.2. Training the Fusion System

After the training process for all the models is finished, the resulting segmentations for the training dataset are saved to disk. The model-generated masks are needed for this step, and running inferences on the network is much more time-consuming than loading the images from memory. 

We defined the Fusion model in the PyTorch framework as an extension to the base Module class. This allows us to define custom network parameters and to override the forward method, which runs model inference. The Fusion model can take as input either a batch of 2D images with four channels or a batch of 2D images with one channel. In the first case, the four channels represent the masks generated by each segmentation model. This mode was used for Fusion system training, as it can load the previously generated masks without passing the images through the segmentation networks. The second case is used when a new CT image must be evaluated through the Fusion system. The model internally loads all segmentation models and evaluates their output, which is then processed in the same manner as in the first case. The Fusion system has the role of weighting the masks provided by each segmentation model, then adding them and producing a final mask. To perform this operation, four independent weights can be defined as model parameters, enable gradient tracking, and compute the result in a weighted sum function. However, a much more elegant solution, which takes advantage of the convolution mechanism provided by the PyTorch framework, is to compute this weighted sum through a convolutional layer. The layer takes an input with four channels, computes the convolution with a 1 × 1 kernel, and outputs the single-channel image. The trainable parameter of this layer is a tensor of shape (4,1,1,1), essentially four values that weigh each mask accordingly and sum the result into one value. The convolutional layer also has the possibility of training a bias parameter, but this was disabled, since it would add a constant value to each binary mask, which cannot improve performance for this application. This method also takes advantage of the built-in autograd mechanism, allowing us to optimize this parameter in the same way that one would train a traditional model.

Using the same stochastic gradient descent algorithm, the weight corresponding to each segmentation network is updated to minimize the cross-entropy loss between the liver class and the background class. The training dataset consisted of the collection of masks generated by each model as input, and the LiTS17 reference segmentations as ground truth. This optimization process is performed in an iterative fashion over five epochs, with a learning rate of 10 and a momentum of 0.9. Since this model has a very small parameter space, the choice of the number of iterations and the learning rate scheduling is less strict. In the experiments, we observed that the four optimized values converged to the same value after roughly four epochs, regardless of the learning rate, if they were in the interval [1,15]. For larger values, the solution had a tendency to oscillate around the optimum. Small values for the learning rate caused the model to converge slower, but the minimum point was approximately the same, within a margin of 10^−5^.

**Figure 9:**
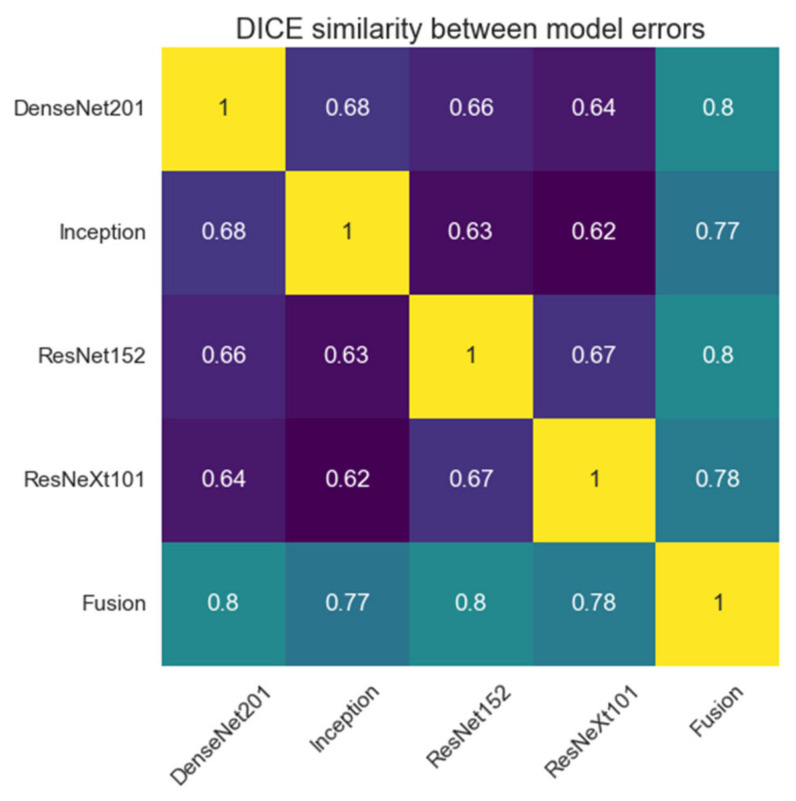
Dice similarity between segmentation errors for liver tissue.

Since the idea of segmentation fusion is that each network will produce errors in different spots in the image, and a decision system could filter out individual errors, the segmentation errors were also calculated as the absolute difference between the ground truth segmentation and the segmentations provided by each network. Then, the Dice similarity coefficient is calculated between every two models over all validation segmentations. A low dice value would be desired since it would mean that the errors do not overlap and have the potential to be eliminated by the fusion system. Figure 9 presents the matrix of the Dice coefficients. Between all models before fusion, the error overlap is under 70% Dice similarity.

### 5.2. System Testing

The Dice score was calculated per examination (i.e., for a whole volume, not for each individual slice) and then averaged over the 10 volumes reserved for testing. Table 1 shows the segmentation scores for each model with and without post-processing, as well as for the fusion system, which was evaluated with the post-processed images. In the case of lesion masks, the proposed post-processing method did not provide a measurable change in performance metrics, so only one set of scores is displayed, as shown in Table 2. As expected, not all models have the same performance, which is influenced by the model’s architecture and the number of parameters. This difference in segmentation performance can also be seen in the proportion each model’s decision has in the result of the Fusion system.

**Figure 10:**
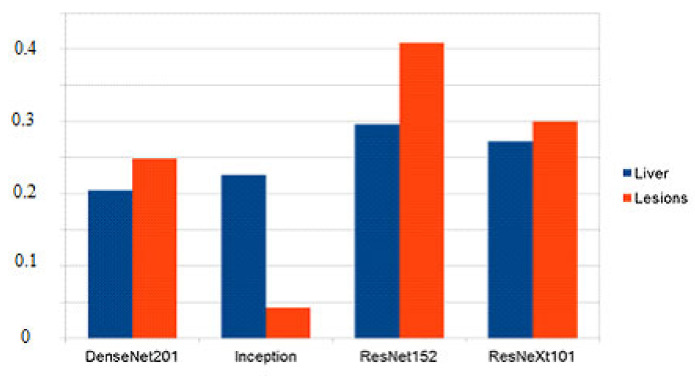
Scaled weights corresponding to each model.

Models which have better performance individually get assigned a higher relative weight, which means that their segmentation has a higher chance to reach the result. Since no model has a weight higher than 0.5 and the final decision is made by rounding the summed and weighted masks, it means that no model decides by itself what the output mask looks like, and at least 2 or 3 of the models must agree on a pixel value for it to be marked as part of the final mask. Starting with equal weights of 25%, the optimized values are as follows for the two fusion systems implemented (Figure 10):

Even though in the case of liver segmentation, the model weights are reasonably balanced since the performance values are closer, in the second case, we can see that the Inception-generated masks have a weight under 5% due to their significantly lower Dice score.

**Figure 11:**
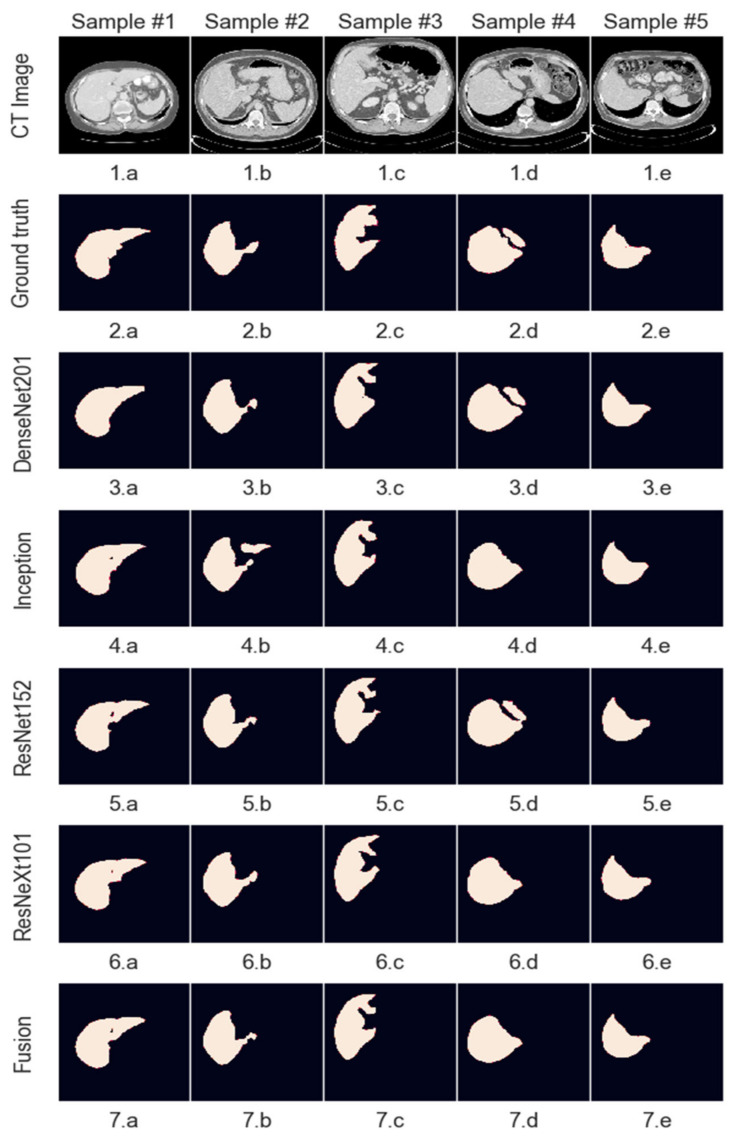
Sample liver segmentations.

**Figure 12:**
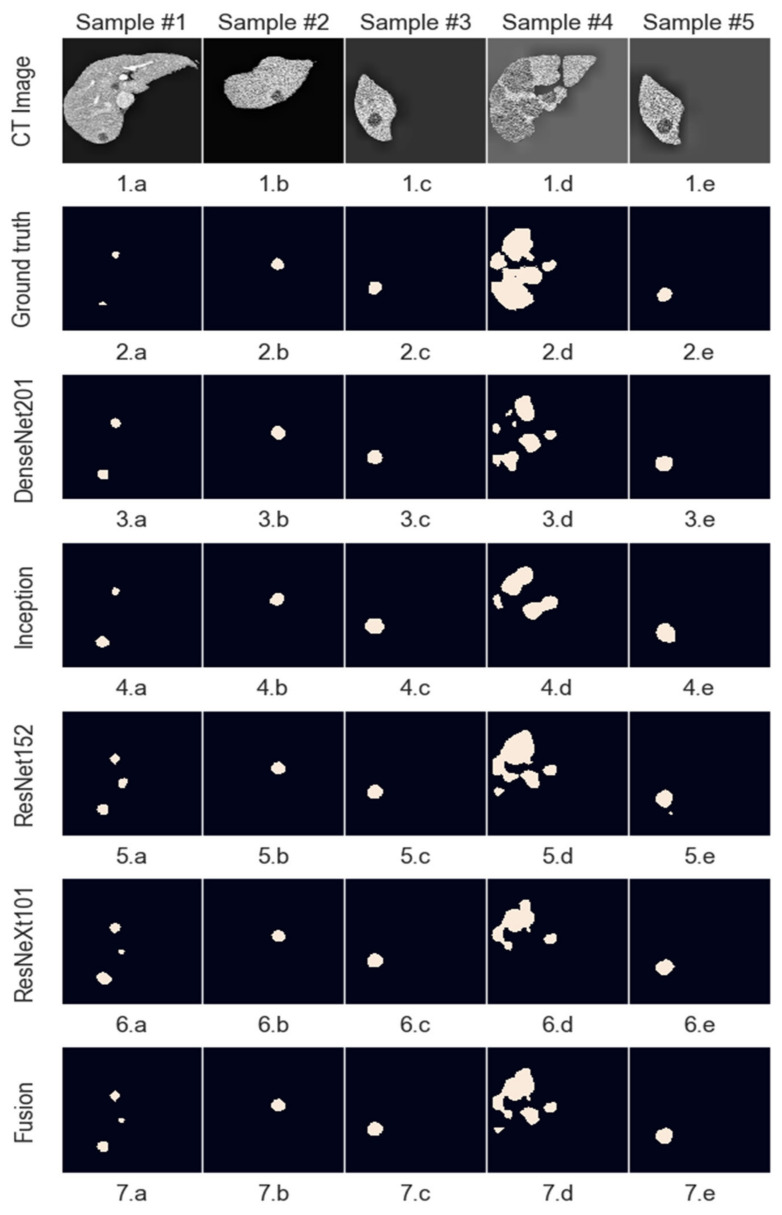
Sample lesion segmentation.

The figures below show a set of randomly picked CT scans from the testing dataset along with their corresponding segmentation and ground truth. The second sample (Figure 11) shows how an incorrect segmentation, which appears only in the case of the Inception network, is removed by the decision fusion system. On the contrary, the fourth sample highlights a case in which two networks segment both liver lesions, while the other identifies only the main area, and the fusion system eliminates the smaller area. This suggests that the post-processing parameters must be adjusted further to ensure that there are no liver regions left out by the segmentations. Figure 12 shows the model outputs for a randomly selected sample of liver images. For cases where the tumor burden is small, and there is only one lesion area, the reference mask, and the fusion mask overlap almost perfectly. On the other hand, as the tumor load increases and more regions appear, some regions are missed by the proposed system. Lastly, darker areas in some images can be falsely detected as lesions, even though they are shadows caused by the X-ray scanning procedure or signify the presence of denser tissue.

The meanings of the notations in Figure 11 and Figure 12 are the following: line 1—initial images obtained from CT, line 2—reference segmentations (ground truth), line 3—results obtained with the individual network ResNet201, line 4—results obtained with the individual network Inception V3, line 5—results obtained with the individual network ResNet 152, line 6—results obtained with the individual network ResNeXt101, line 7—global decision fusion system; columns a, b, c, d, e—examples of images selected for testing.

**Figure 13:**
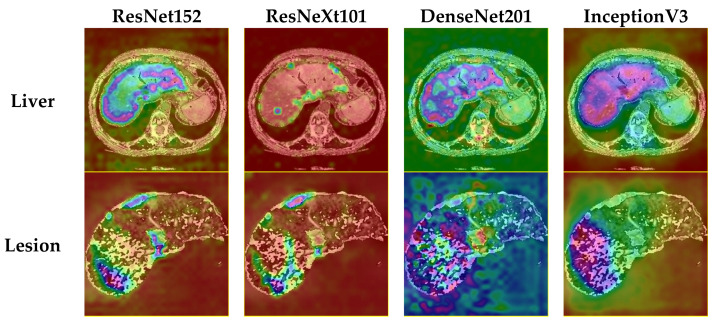
Feature extractor activations for each individual model.

To further emphasize the localization capabilities of the classification models that we used for feature extraction, we generated activation maps for the last convolutional layer of each segmentation network backbone (Figure 13). These results show that, even before the semantic segmentation stage, the classification structure shows intense activation in the areas of interest. One notable difference appears in the case of ResNeXt, where the layer output activates only on the liver outline and not on the whole area. In the case of lesion segmentation, only the residual models showed an accurate location for the affected areas.

## 6. Discussion

In this paper, we have proven that multiple image classification convolutional neural networks can be used as feature extractors for semantic segmentation in the context of medical imaging, specifically liver segmentation, and that a decision fusion system can further improve the performance of these networks by removing individual errors. Model performance was also increased by employing various pre-processing and post-processing techniques on the images and the resulting masks. From the results and the performance metrics obtained, we deduce that this solution produces satisfactory results but still needs adjustments and parameter fine-tuning to be on par with state-of-the-art methods, such as improved UNets. 

Comparing the proposed method with state-of-the-art solutions, some improvements still must be made. The comparison table (Table 3) shows that some methods based on more advanced variations of the UNet architecture achieve better performance metrics. It should be noted that most of the methods in Table 3 (see references [14,16,19,20,34]) are based on a 3D (volumetric) approach, which is much more expensive in terms of memory and resources than the method proposed by us.

Also, when discussing performance, model size should also be considered. To achieve these scores, we used variations in classification networks that have a high number of layers when compared to encoder–decoder networks. For example, for the proposed system, the size of all model weights stored on disks reaches almost 1800 MB, while UNets occupy a size around 30 MB for the base variant and up to 120 MB for more complex variations. This means that the Fusion system, along with its internal models, is a lot slower to load, and needs significantly more computational resources to train; the inference times are also higher. However, as this is a medical application, there is no need for instant segmentation results, so high processing times are not necessarily a significant drawback.

Neural networks used as individual classifiers can be considered elements of subjective intelligence. Grouped in a system of merging decisions, the latter can be seen as collective intelligence. There are several problems that need to be solved: What is the set of neural networks used? How many neural networks will be used in the system? A compromise must be made between the complexity of the global system and efficiency based on statistical indicators and the learning/testing time. It is necessary to analyze the way of considering individual decisions in global decision-making. Obviously, the classic voting method is outdated. Sometimes, compensatory decisions should also be considered. It cannot be considered a priori that an increase in the number of neural networks automatically leads to an increase in classification or segmentation performance. It will obviously reach saturation. The proposal of our system was based only on experimental results, without such analyses being made based on expert opinions or biopsies, liver resection, or transplant specimens.

In the future, we would like to extend our work to MRI imaging of the liver. Considering the increased specificity and sensitivity of using MRI and especially intracellular contrast agents (Gadoxetate disodium) compared to contrast-enhanced CT for liver lesion detection and characterization [37], our idea is to create a system capable of segmentation and detection of liver and hepatic lesions on hepatobiliary phase imaging. Other authors have published works on hepatospecific MRI imaging deep learning tools [38], and these, together with our current work, will provide a starting point for the development of this model.

## 7. Conclusions

The proposed system for liver and tumor segmentation is a multi-network structure with better performance than the networks that make it up. The system contains four deep neural networks as individual classifiers and a perceptron as the final decision module. The system structure and the proposed multi-phase training method are the main contributions. Although implemented on a simpler and less expensive structure than 3D systems, the performance of our system is similar. In the future, a theoretically grounded solution for choosing component networks will be considered to obtain maximum performance with a minimum cost. In future work, after the system parameters are adjusted to match the needs in clinical settings, the next step would be to devise an artificial intelligence system that can analyze the identified tumor patches across multiple CT contrast phases. Based on tissue behavior across these phases, the specific type of lesion can be theoretically identified. However, to achieve this, a separate database needs to be created, one that contains lesion classifications based on expert opinions or biopsies, and which also contains examinations corresponding to the three contrast phases for all patients. A system that could provide a specific diagnosis based on these CT scans would bring great value in clinical settings, as costly and invasive biopsies could be avoided in some cases. Reliable automated diagnostics systems still have a long way to become independent of expert opinion, based partly on the fact that there is great variability in the human body that cannot be captured by a medical image dataset, regardless of how large it is.

## Figures and Tables

**Table 1:** Dice scores for liver segmentation.

Model	InceptionV3	DenseNet201	ResNet152	ResNeXt101	Fusion
Without post-processing	94.41%	94.94%	95.54%	95.28%	-
With post-processing	95.04%	95.25%	95.59%	95.27%	95.67%

**Table 2:** Dice scores for tumor segmentation.

Model	InceptionV3	DenseNet201	ResNet152	ResNeXt101	Fusion
Dice score	57.38%	70.15%	75.12%	74.61%	75.83%

**Table 3:** Performance comparison between the proposed system and other works.

Method/Dimension	Reference/Year	Liver	Lesion
residual UNet/2.5D	[18]/2017	-	67%
UNet + graph cut/2D	[13]/2019	95.05%	-
TDP-CNN/3D	[14]/2020	96.50%	68.9%
3D RA-UNet/3D	[16]/2020	96.10%	-
SAR-UNet/2D	[15]/2021	95.71%	59.5%
2.5D UNet/similar3D	[19]/2021	95.30%	78.4 ± 16%
XNet/3D	[20]/2021	97.10%	84.3%
3D U-Net/3D	[34]/2022	94.20%	
CEDRNN/2D	[35]/2022	95.20%	-
EfficientNet and Attention-Based Residual U-Net/2D	[36]/2022	95.20%	-
Decision fusion/2D	our	95.76%	75.83%

## Data Availability

Publicly available datasets were analyzed in this study. This data can be found here: 1. https://competitions.codalab.org/competitions/17094 (accessed on 1 August 2022), 2. https://www.ircad.fr/research/data-sets/liver-segmentation-3d-ircadb-01/ (accessed on 1 August 2022).
